# Extra-abdominal desmoid fibromatosis: An evaluation of clinical factors affecting local recurrence rates

**DOI:** 10.5152/j.aott.2021.21033

**Published:** 2021-11-01

**Authors:** Hüseyin Sina Coşkun, Furkan Erdoğan, Hikmet Çinka, Nevzat Dabak

**Affiliations:** Department of Orthopedics and Traumatology, Ondokuz Mayıs University, School of Medicine, Samsun, Turkey

**Keywords:** Local recurrence, Desmoid fibromatosis, Surgery/Radiotherapy

## Abstract

**Objective:**

The aim of this study was to determine the prognostic factors for recurrence in patients with extra-abdominal desmoid tumors (EDTs) treated surgically.

**Methods:**

This single-institution, retrospective study included patients with a histologically-proven extra-abdominal desmoid tumor between 2007 and 2018. The demographic characteristics (age, sex) of the patients, tumor characteristics (region, size, proximity to neurovascular structures, margins), treatment management (surgery and/or adjuvant radiotherapy), and clinical results were analyzed. The effects of these possible prognostic factors on overall and disease-free survival rates and the risk of local recurrence were evaluated.

**Results:**

Evaluation was made of 22 patients (16 females, 6 males) with a mean age at diagnosis of 34.7 years (range = 22-76 years). The mean follow-up was 104 months (range = 4.8-168). Tumor localization was in the upper extremity in 4 patients (18.1%), the lower extremity in 11 (50.0%), and the trunk in 7 (31.8%). The mean tumor size (maximum diameter) was 5.2 cm (range = 0.6-13 cm; median = 5.8 cm), and the mean tumor volume was 181.3 ± 531.4 ml. All the 22 patients were treated surgically along with adjuvant radiotherapy (RT) administered to 8 in addition to surgery for the primary treatment of the tumor. Following primary surgery, resection margins were R0 in 11 patients, R1 in 9 and R2 in 2. Local recurrence (LR) developed in 6 patients (27.2%) during the follow-up period. Recurrence-free survival rate (RFS) was 90.9% at one year, 74.1% at 5 years, and 61.7% at 10 years. During the follow-up, no patient died, and distant metastasis was not detected. Tumor length, resection margins, and adjuvant RT were observed to influence the risk of local recurrence (*P* < 0.05).

**Conclusion:**

The results of this study have demonstrated that tumor size ≥ 5 cm and the presence of microscopic or macroscopic positive surgical margins can increase the risk of LR, and adjuvant RT can reduce the development of LR in the management of EDT.

**Level of Evidence:**

Level IV, Therapeutic Study

## Introduction

Desmoid tumor or aggressive fibromatosis is one of the soft tissue tumors that do not metastasize but show locally aggressive features, which can occur in many parts of the body. These tumors arising from connective tissue in the muscle, fascia, or aponeurosis are rare, with a reported incidence of approximately 2-4 per million per year,^1^ and account for 3% of all soft tissue tumors.^2^ Extra-Abdominal desmoid tumors (EDTs) frequently appear as nodular or bulky masses. However, these tumors can be detected macroscopically or microscopically, with a tentacle-like spiculate pattern with infiltrative growth.^3^

There is no consensus on the optimal treatment of Extra-Abdominal Desmoid Tumors (EDTs), so no protocol for optimal treatment management has been established. Extensive surgical resection is considered, the standard treatment for EDTs for reasons such as the negligible rate of recurrence and difficulty in determining the true size of the tumor.^4,5^

In addition to surgery, various systemic treatments may be used, including Radiotherapy (RT) and chemotherapy, radiofrequency ablation and cryotherapy, hormone therapy, or nonsteroidal anti-inflammatory drugs.^6,7^ There are also reports in the literature that support the wait-and-see policy instead of surgery as the first step in management.^8^

When the current literature is reviewed, there can be seen to be controversy about the effects of potential prognostic factors, such as resection margin, tumor size, and adjuvant RT, on Local Recurrence (LR) risk and disease-free survival after treatment.^9,10^

The aim of this study was to investigate the effect on the LR of extra-abdominal desmoid tumor of prognostic factors such as age, resection margin, tumor location and size, and adjuvant RT use.

## Materials and Methods

**Figure 1.**
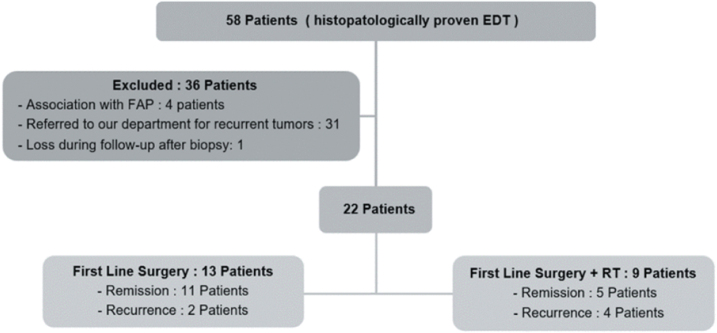
Process for patient selection and treatment management for the study.

The study included 22 patients of all ages with primary EDT located in the trunk and extremities, who were treated in our institute between May 2007 and February 2018. None of the patients had a history of LR of desmoid tumor and therefore no previous surgery. Patients were excluded if they had received drug regimens with previous neo-adjuvant therapies for EDT, had a history of surgery for recurrence, or had received RT associated with familial adenoid polyposis (Figure 1).

Data were retrieved from patient files in respect of age at the time of diagnosis (pediatric ≤ 18 years, adult > 18 years), tumor localization and size, the margin of resection, the administration of adjuvant RT, follow-up period, recurrence during follow-up, and if so the time from surgery. Recurrence-Free Survival (RFS) was calculated, and the relationship of these possible prognostic factors with LR was investigated.

Magnetic Resonance Imaging (MRI) was performed in all patients at the time of diagnosis, and tumor size (including the largest diameter) and volume were measured. According to the mean tumor size, the cases were separated into two groups with a cut-off value of 5 cm.^11,12^ The proximity of the tumors to neurovascular structures was examined. Microscopic margin status was obtained from the final pathology reports issued by a single pathologist, and data were retrospectively reviewed. The results were grouped as R0 (wide resection without even microscopic tumor residue), R1 (microscopic tumor positive margins), or R2 (macroscopic residual disease) using the International Society against Cancer (UICC) classification.

Adjuvant RT applied to patients in this study was planned and implemented by the multidisciplinary tumor council according to the National Comprehensive Cancer Network (NCCN)^13^ guidelines. Patients with large masses in critical areas such as the shoulder and hip, cases with gross residual mass (R2), and cases with LR were given RT at a dose of 56 Gy as 2 Gy per fraction in 5-7 weeks. No neoadjuvant or adjuvant medical treatment was given to the patients, except 150 mg of Diclofenac sodium daily in divided doses.

### Statistical analysis

Data obtained in the study were analyzed statistically using the Statistical Package for Social Sciences (SPSS) version 22.0 (IBM SPSS Corp., Armonk, NY, USA). The Chi-square or Fisher’s exact test was used to compare categorical variables. The disease-free survival rates of the patients were determined using the Kaplan-Meier method and the comparisons were analyzed with the Log-rank test. Univariate and multivariate analyzes were performed using Cox regression analysis. A value of *P* < 0.05 was considered statistically significant.

### Ethical approval

Approval for the study was granted by the Clinical Research Ethics Committee of our Institute on 29.06.2020 with the decision number 2020/444.

## Results

An evaluation was made of a total of 22 patients diagnosed with EDTs between 2007 and 2018. The patients comprised 16 (72.7%) females and 6 (27.3%) males with a mean age of 34.7 years (range, 22-76 years) and a mean follow-up period of 104 months (range, 4.8-168 months). The patients were separated into pediatric (<18 years) and adult (>18 years) age groups. There was no significant difference between the two groups (*P* = 0.110). There was also no significant relationship between gender and recurrence (*P* = 0.501). The characteristics of the 22 patients are detailed in Table 1.

The patients were separated into three groups according to tumor location: upper and lower extremities, and trunk. The most common locations of the tumors were the paravertebral region in 7 patients (31.8%), hip-thigh in 6 patients (27.2%), and in the popliteal-calf region in 5 patients (22.7%). LR rate according to tumor location was most common in the lower extremity and then in the upper extremity groups, respectively. The lowest rate was in the trunk, with no statistical significance determined (*P* > 0.05). When the proximity of tumors to neurovascular structures was examined, there was determined to be proximity to paravertebral structures in 6 patients and popliteal and femoral artery proximity in 4 patients. There was no significant difference in terms of LR (*P* > 0.05).

The mean tumor size (maximum diameter) was 5.2 cm (median, 5.8 cm; range, 0.6-13 cm). The cases were separated into two groups according to the mean tumor size with 5 cm cut-off values. Patients with a tumor size ≥ 5 cm were found to be associated with a higher probability of recurrence (*P* < 0.05). Mean tumor volume was 181.3 ± 531.4 mL. In univariate analysis, a cut-off value of 320 mL was determined, and values above this volume were found to be associated with a higher probability of recurrence (*P* < 0.05).

Surgical excision was performed in all patients in the primary treatment of the tumor, and adjuvant RT was added to the treatment of 8 patients (41%). The characteristics of primary treatment and the surgical parameters are presented in Table 2.

The RFS rate was 90.9% (95% confidence interval [CI], 75-97%) at 1 year and 74.1% (95% [CI], 61-87%) at 5 years. The RFS rate was 67.1% at the final follow-up examination after an average of 9.6 years. None of the patients died because of disease, and no distant metastases were detected during the follow-up period.

A total of 11 LRs developed in 6 patients (27.2%), as 1 in 2 patients, 2 in 3 patients, and 3 in 1 patient. The mean time from surgery to recurrence was 3.1 years (range, 0.25-12 years). Of the 6 patients who received adjuvant RT following the first surgery, 4 (66%) developed LR, all of which were in the region previously exposed to radiation. Of these 4 patients, 2 had R1 resection margins in primary surgery, and 2 had R2. After the first LR, surgical treatment was applied only to the 2 patients who had not previously received RT, and surgical treatment + adjuvant RT was applied to the other 4 patients. During the follow-up period, a second recurrence was observed in 4 of 6 patients who were treated after the first LR.

Surgery + RT was applied to all these patients who developed a second recurrence. After 18 months, a third LR was detected in one of these patients. This patient, who developed LR for the third time, had been given RT with surgical treatment due to gluteal localization on the first admission, and was applied surgical treatment + RT for the third time. In total, recurrence was seen in 4 patients in the R2 resection group, 5 patients in the R1 group, and 2 patients in the R0 group. All the patients with recurrence were followed up without disease. The data of the patients with recurrence are summarized in Table 3.

In the evaluation of the tumor margin groups, one of 11 patients (50%) who had wide resection (R0) developed LR, and a second and third operation was performed to obtain clear resection margins microscopically. Secondary surgery + RT was performed in 3 (13.6%) of 9 patients with a result of microscopically incomplete resection (R1) due to the development of LR during follow-up. Recurrence was observed in all the patients in the R2 group (9%) during follow-up, all of which were treated with surgery + RT. LR development was found to be significantly lower in the R0 group than in the other groups (*P* = 0.03).

The patients who received postoperative RT were found to have a 40% lower LR rate after RT compared to the group who did not receive RT. In the multivariate analysis, RT was determined to be effective in preventing LR (*P* < 0.05).

## Discussion

This study summarizes the experience of a single institution in a little more than a decade of treatment and presents the clinical outcomes of EDT patients. In the light of these clinical results, some prognostic factors with potential impact on LR were examined and compared with the results of similar studies in the literature.

The main treatment for desmoid tumors is surgical excision. The main purpose of surgical treatment should be to achieve the best possible functional and cosmetic results and to reach negative surgical margins. Adjuvant RT may be added to the treatment in selected cases. Although it has been reported in the literature that distant metastases are almost never seen in the postoperative follow-up of these tumors, the high LR rate stands out as the main failure pattern.

Similarly, in the current study, LR occurred 11 times in 6 cases, and the 5-year risk of LR was seen to be at an average level compared to the data reported by other researchers in the literature. A previous systematic review reported that relapse rates ranged between 6% and 59%.^14^

The relationship between age and prognosis in EDT is controversial. Although many studies have reported that younger age in desmoid tumors is a predictive factor for shorter RFS after surgical treatment,^15,16^ there are also publications showing that age has no effect on LR.^17^ In the current study, when the patients treated with surgery and RT were separated into pediatric and adult age groups, it was determined that age and gender had no effect on LR.

In the current study, tumors ≥ 5 cm in size were determined to have a higher recurrence rate and were associated with LR. Although a clear surgical margin was obtained after resection, especially in a patient with a mass > 12 cm, the development of 3 LRs during follow-up supports this conclusion. Therefore, tumor size can be considered to be a possible risk factor for poor prognosis.

Although tumor size has been adopted as a known independent prognostic factor, previous studies have shown controversial results.^9,11,18^ Some authors have emphasized that the larger the tumor size, the more aggressive it can become and the greater the LR rate. He et al.^19^ reported that tumor size > 8 cm increases the risk of LR, and suggested that large tumors may be anatomically close to vessels and nerves and therefore difficult to remove with clear margins. In contrast, there are reports stating that there is no independent risk factor in terms of tumor size and prognosis.^11,20^

Although it was found in the current study that tumor volume > 320 mL increased the risk of LR, there are reports in the literature which have evaluated volume after medical or other treatment rather than surgical treatment.^21,22^ Those studies have presented conflicting results in the form of measuring the volume of tumors and evaluating the effect of treatment according to the volume change. Therefore, the relationship between volume and LR is an unexplored issue that needs to be addressed in future studies.

Less LR was observed in the current study patients who had R0 surgical resection margins compared to the other groups. There are conflicting results about surgical resection margins from previous reports. In some retrospective studies, in line with the current study findings, better long-term local control has been reported after R0 resection.^23,24^

In a recent cohort study, R2 resection was associated with a higher risk of tumor recurrence, but a microscopically tumor-free resection margin (R0 resection) was not associated with better local control than R1 resection.^9^ However, in larger studies,^13^ margin status has not been shown to have any effect on recurrence. However, the differing results between surgical margin and recurrence may be due to poor detection of possible spiculated and infiltrative extensions in areas more distant from the main tumor. In this respect, there are reports stating that high-resolution ultrasonography (HRUS) may have additional advantages over MRI in the diagnosis of tumors.^25^

There can be considered to be a need for long-term follow-up to be able to understand the consequences of resection margins on LR.

Although there was no difference in LR in patients who received postoperative RT compared to those who did not receive RT, the current study results showed that postoperative RT significantly reduced the risk of LR.

There are various studies in the literature with different results on whether postoperative RT is beneficial in patients with microscopic or macroscopic residual tumors and recurrent disease. Nuyttens et al.^26^ evaluated 381 patients treated with surgery alone and 297 patients treated with surgery + adjuvant RT and concluded that the addition of RT in the postoperative period had a significant effect in reducing recurrence and its effect in reducing LR after R1 and R2 resection was particularly strong. In contrast, some large-scale retrospective studies,^13,18,27^ have stated that adjuvant RT does not have a significant effect on recurrence and its use should be questioned.

In the current study, adjuvant RT was found to reduce the risk of LR after surgical resection in patients with positive resection margins and large tumors (mean tumor length of 8.3 cm). The role of RT in local control of the tumor has not been clearly defined in the literature, and its place in treatment remains unclear. However, concerns about the potential late effects of radiation still surround its use.

As a total of 11 LRs in 6 patients with significant vascular nerve proximity of the tumor and LR in 6 of 10 patients, most of the LRs in the postoperative period were observed on the basis of the previous recurrence. This could be attributed to the size of the tumor, the extension of the mass along the fascial planes between the muscle fiber bundles, and the inability to preserve the surgical margins during tumor excision close to the neurovascular structures. In addition, LR may have been caused by factors that we previously evaluated, patient-related factors, or unknown tumor-related factors. However, although neurovascular proximity has not been found to have a statistically significant effect on recurrence, it can be accepted that the technique used in primary surgical treatment is a possible prognostic factor in terms of LR.

Interestingly, one patient who was excised microscopically with a tumor-free resection margin (R0) and was followed up for a long period without disease after surgery, recurrence was detected in the same site after 12 years. This patient, who had no familial adenomatous polyposis or similar history, received RT to the region after re-operation. Therefore, it can be seen to be necessary to be alert during the follow-up by providing adequate information to the patient about the LR rate of this tumor.

All the patients in this study were primary cases and had no history of treatment at an external center. Data on other important parameters affecting recurrence rates, such as patient age, tumor size, and precise anatomic location, were fully obtained. The treatment of the patients was applied in a single center and by the same surgeon, and homogeneity was achieved in the patient and treatment groups as a result of the treatments performed with the same management protocols. The fact that these patients had a follow-up period of more than 10 years in a single center is the strength of this study in terms of follow-up complications.

The limiting factors of our study were the relatively limited number of patients treated and therefore unhealthy results in statistical analysis. Therefore, the inferences made here may not be valid for other patient groups. The strength of our study is that our analysis can support the literature since it is a rare entity.

In this study, most of the patients with LR had recurrence during postoperative follow-up, which may have been due to factors previously evaluated or to unknown patient-related or tumor-related factors. Although the limited sample size of this series makes it difficult to reach precise guidelines on the treatment of desmoid tumors, it appears that wide resection provides significantly lower recurrence rates than surgical procedures with intralesional or microscopic surgical margins. However, it can be concluded that postoperative RT use decreases the risk of LR and benefits patients with recurrent disease in near or microscopically positive margin resections. In conclusion, obtaining tumor-free margins in surgery and using RT in near or microscopically positive margin resections are important within the framework of a multidisciplinary approach in the management of desmoid tumor treatment.

**HIGHLIGHTS**

- Primary surgical operation can be considered an important factor for prognosis in terms of LR for extra-abdominal desmoid fibromatosis.
- Surgical margin positivity has been consistently associated with an increased risk of LR, and wide resections with R0 margins are generally associated with lower LRs.
- In this study, tumor size ≥ 5 cm and volume > 320 mL was determined as a risk factor for the development of LR. Which implies that larger tumors may have a worse prognosis.
- Postoperative irradiation may be beneficial in patients with microscopic or macroscopic residual tumors and recurrent disease.

## Figures and Tables

**Table 1.** Patient Characteristics

Categories	Overall	%	RFS (Recurrence Free Survival) *p*
Age (mean years)	22		
Pediatric ≤ 18 years	8	36.4	0.10
Adult > 18 years	14	63.6	
Sex (no. of patients)			
Male	6	27.3	0.48
Female	16	72.7	
Follow-up period (months)	104	4.8-168	
Primary anatomic sites			
Upper limb	4	18.1	0.81
Lower limb	11	50.0	
Trunk	7	31.8	
Location			
Shoulder and Arm	4	18.1	0.93
Paravertebral region	7	31.8	
Hip and Thigh	6	27.2	
Poplitea and Calf	5	22.7	
Tumor size (cm)			
0-5	9	40.9	0.02
≥5	13	59	
Tumor volume (mL)			
0-320	4	18.2	0.01
> 320	18	81.8	
Margin status			
R0 - Wide margins	11	50.0	0.03
R1 - Microscopic residual disease	9	40.9	
R2 - Macroscopic residual disease	2	9.0	
Radiation treatment			
Yes	8	36.3	0.02
No	14	63.6	
Adjacent to nerves/vascular structures			
Yes	10	45.4	0.15
No	12	54.5	
Recurrence			
Yes	6		
No	16		

**Table 2.** Primary Treatment and Surgical Margins

Categories	Surgery	%	Surgery + Adj RT	%
Primary treatment management	13	59.0	9	41.0
Localization of the tumor				
Upper extremity	1	4.54	3	13.6
Lower extremity	6	27.2	5	22.7
Trunk	6	27.2	1	4.54
Size of the tumor (cm)				
0-5	7	31.8	3	13.6
≥ 5	6	27.2	6	27.2
Surgical margins				
R0 - Wide margins	10	45.4	0	0
R1 - Microscopic residual disease	3	13.6	7	31.8
R2 - Macroscopic residual disease	0	0	2	9.09

**Table 3.** Surgical Data of Patients with Recurrence

Categories	Primary LR	%	Other LRs	%
Treatment after primary recurrence	6	100	5	100
Surgery + Adjuvant radiotherapy	4	66.6	4	75.0
Only surgery	2	33.3	1	25.0
Surgery + RT				
Outside area of RT	0	0	0	0
Inside previous RT field	4	100	4	100
Prior Surgical margins (Surgery + RT)				
R0 - Wide margins	0	0	1	0
R1 - Microscopic residual disease	2	25.0	2	25.0
R2 - Macroscopic residual disease	2	25.0	2	25.0
Prior Surgical margins (Only surgery)				
R0 - Wide margins	1	7.1		
R1 - Microscopic residual disease	1	7.1		
R2 - Macroscopic residual disease	0	0		
